# Unusual stent after ureteral substitution. A first case

**DOI:** 10.1186/1471-2490-12-34

**Published:** 2012-11-29

**Authors:** Girolamo Fiorini, Giorgio Pomara, Francesca Manassero, Andrea Mogorovich, Lorenzo Faggioni, Cesare Selli

**Affiliations:** 1 Department of Urology, University of Pisa, Via Paradisa 2, 56100, Pisa, Italy; 2 Diagnostic and Interventional Radiology, University of Pisa, Via Paradisa 2, 56100, Pisa, Italy

**Keywords:** Ureteral stent, Ileal loop substitution, Abdominal X-ray, Multidetector computed tomography, Image processing

## Abstract

**Background:**

To the best of our knowledge this is the first case where a Silastic drain is used in ureteral surgery instead of a common urological stent. Patients coming from other institutions, especially in peripheral areas, can be treated with non conventional devices and if traditional imaging is inconclusive, computed tomography (CT) can provide valuable information to make the right diagnosis.

**Case presentation:**

We present the unusual case of a 32F Silastic drain found inside the urinary tract in a female patient who had previously undergone ileal loop replacement of the left ureter for post-hysterectomy stricture at another Institution, and had subsequently repeated surgery due to persistent hydronephrosis. Radiological findings on plain abdominal X-ray were quite misleading, while CT allowed a correct assessment of the drain features.

**Conclusion:**

While double J stents of different lengths, sizes and materials are used in ureteral surgery, the use of Silastic drains has not been previously reported. In light of the present experience we don’t suggest its routinely use.

## Background

While double J stents of different lengths, sizes and materials are used in ureteral surgery, to the best of our knowledge the use of Silastic drains has not been reported. This uncommon finding is due to the fact that the procedure had been performed by General Surgeons, and is also explained by the size of the intact ileal loop. The poor outcome of bowel segments incorporated into the closed urinary system was reported first more than 30 years ago [1], and at present, only tapered ileum is used for ureteral substitution (Yang-Monti procedure) [2,3].

Plain radiography of the abdomen is the diagnostic procedure most commonly performed to assess the position of ureteral stents. Its main advantages are its low cost, technical ease, ubiquitous availability even in small hospitals, very fast imaging time, and relatively low radiation dose. However, while interpretation of X-ray images is usually straightforward in patients with correctly positioned or (at the opposite extreme) grossly displaced ureteral stents, findings can be inconclusive or misleading when nonconventional devices are used or in case of subtle rupture or displacement of ureteral stents [4-6]. In such situations, computed tomography (CT) can provide valuable information that may be vital to make the right diagnosis. In particular, the currently widespread availability of multislice CT scanners with up to 64 detector rows and even more allows to acquire in a few seconds a series of cross-sectional images of the entire urinary system with slice thickness less than 1mm [7-9]. This is essential to get a detailed depiction of the urinary tract and ureteral devices, either using native axial sections or bi- and three-dimensional views, that can be generated in post-processing without delivering any additional radiation dose to the patient to highlight specific details of the anatomy under investigation [9-11].

In this article we report an unusual case of a 32F Silastic drain found inside the urinary tract in a female patient who had previously undergone ileal loop replacement of the left ureter for post-hysterectomy stricture at another Institution. Radiological findings on plain abdominal X-ray were quite misleading, while CT allowed a correct assessment of the drain features.

## Case presentation

A 59-year-old woman was admitted for recurrent urinary tract infection (UTI). Past history revealed right hip prosthesis and hysterectomy for cervical cancer at age 56, followed by radiotherapy. An extensive stricture of the left ureter had been treated with ileal loop substitution in a General Surgery environment at age 58. The procedure was performed in a general surgeon setting instead of a urologist because the patient lives in a peripheral area with first level hospitals. Three months later, due to worsening of left hydronephrosis, the patient underwent pyeloplasty with placement of a 'stent' of unknown characteristics using a flank approach. Ten months after the second procedure, the patient was evaluated at our institution for recurrent UTI and fever, with irritative bladder symptoms (frequency and urge incontinence). Serum creatinine was 1 mg/dL; urine culture revealed >1 million E. coli CFU (Colony Forming Units) per mL.

**Figure 1:**
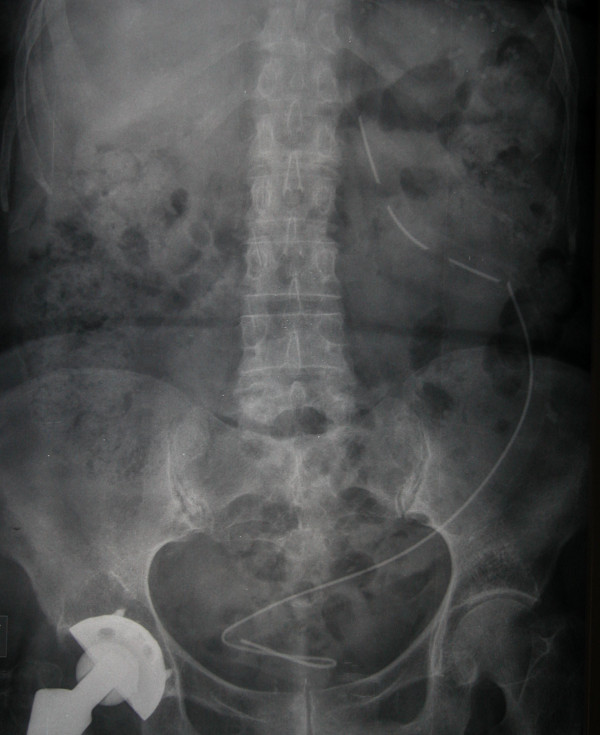
Abdominal X-ray showing a radiopaque stent in the left collecting system: the upper extremity is apparently uncoiled and there are two short interruptions at the proximal third, while the lower extremity forms a wide coil inside the bladder.

As a second step a plain X-ray of the abdomen was performed (Figure 1), that showed presence of a stent-like radiopaque device on the left side, with its upper part projecting on the left kidney area and three short interruptions at its proximal third. Furthermore, a renal scintigraphy was performed, that revealed severely reduced (10%) left kidney function.

**Figure 2:**
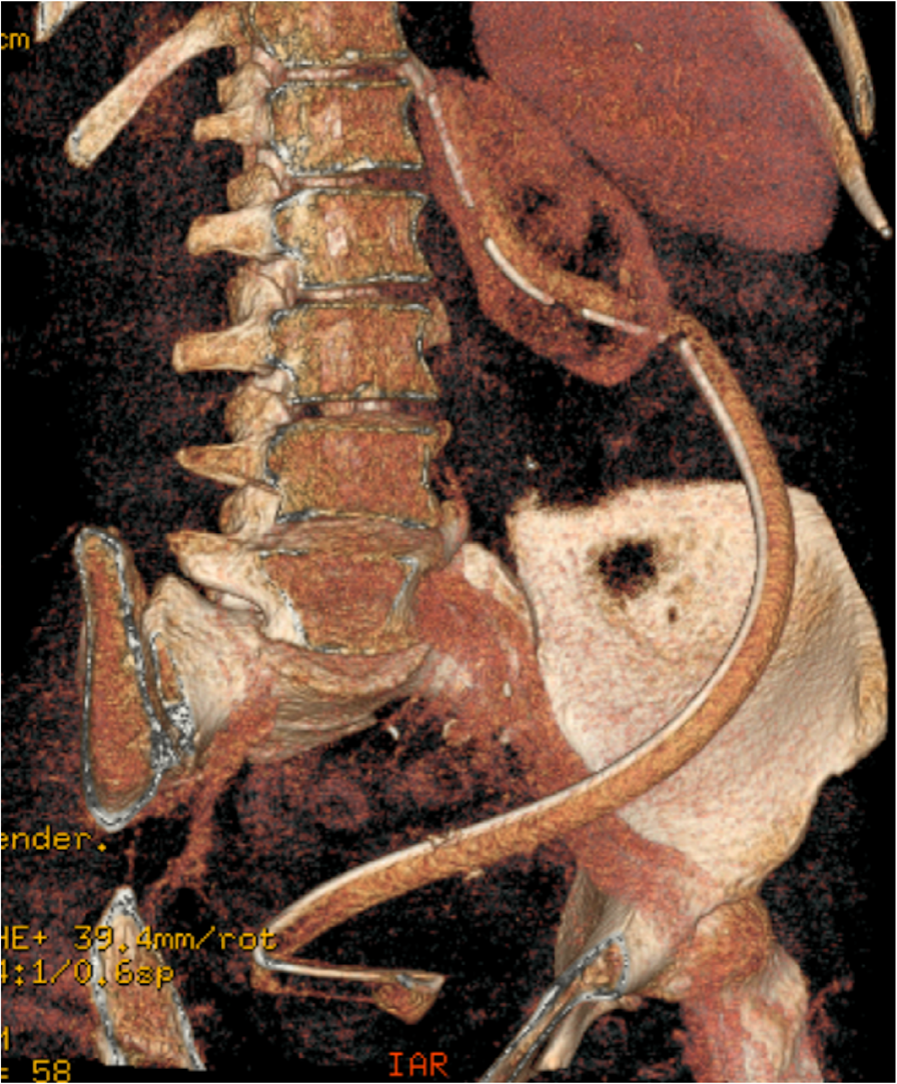
VR reconstruction Multidetector CT urography showing a large tubular structure with a radiopaque marker in the left collecting system.

In order to get more detailed information about the morphology of the left urinary tract, CT urography was subsequently carried out using a 64-row CT scanner. Axial images, Maximum Intensity Projection and Volume Rendering (VR, Figure 2) reconstructions showed instead a large tube with multiple bends and a radiopaque marker inside it, crossing through the entire collecting system of the left kidney; this latter was smaller than the contralateral one, due to chronic parenchymal failure.

**Figure 3:**
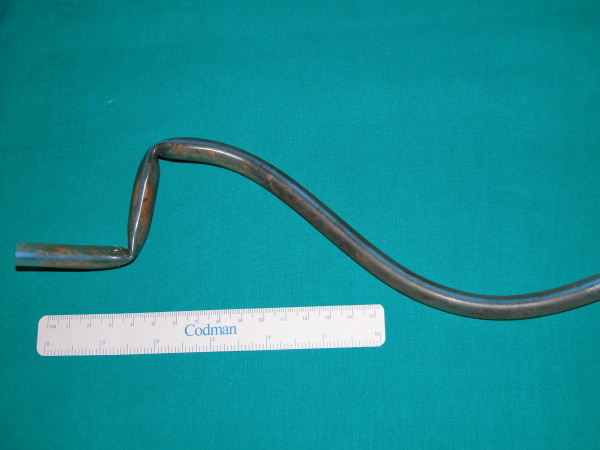
32F Silastic drain removed at surgery with moderate incrustation, placed 8 months earlier.

The patient underwent left nephrectomy and removal of the ileal loop, containing a 32F partially encrusted Silastic drain with a radiopaque marker (Figure 3). The general surgery setting can, even partially, explain the use of a Silastic tube instead of a standard urological stent.

In our case, CT proved to be superior to plain X-ray due to its higher contrast resolution and its ability to display a body volume as a stack of cross-sectional images, rather than as a projective representation of the attenuation of all tissues crossed by a relatively wide X-ray beam [7,8]. Moreover, CT allowed to accurately distinguish the Silastic drain tube from its centrala radiopaque marker, while 2D and 3D reconstructions (and especially, VR) yielded an accurate depiction of the tube structure and course. In particular, MIP views were useful to show the metallic marker in its entirety (by extracting voxels with the highest intensity inside the slab), while VR were more suitable to display the relatively hypodense tube coating, owing to the VR capability to use the information from all voxels for generating a 3D view of the anatomy under investigation [8-11].

## Conclusions

While double J stents of different lengths, sizes and materials are used in ureteral surgery, the use of Silastic drains has not been previously reported. In light of this experience we don’t suggest its routinely use.

### Informed consent

Written informed consent was obtained from the patient for publication of this case report and any accompanying images. A copy of the written consent is available for review by the Series Editor of this journal.

## Competing interests

The authors declare that they have no competing interests.

## Authors’ contributions

GF-GP contributed to conception, design, acquisition and drafting of the manuscript. FM and AM contributed in drafting the manuscript. SF contributed to acquisition of radiological information. CS contributed in drafting the manuscript and revising it critically for important intellectual content. All authors read and approved the final manuscript.

## Pre-publication history

The pre-publication history for this paper can be accessed here:

http://www.biomedcentral.com/1471-2490/12/34/prepub
